# A Seaweed Extract-Based Biostimulant Mitigates Drought Stress in Sugarcane

**DOI:** 10.3389/fpls.2022.865291

**Published:** 2022-04-28

**Authors:** Lucas Moraes Jacomassi, Josiane de Oliveira Viveiros, Marcela Pacola Oliveira, Letusa Momesso, Gabriela Ferraz de Siqueira, Carlos Alexandre Costa Crusciol

**Affiliations:** Department of Crop Science, College of Agricultural Science, São Paulo State University (UNESP), São Paulo, Brazil

**Keywords:** *Saccharum* spp., *Ascophyllum nodosum*, reactive oxygen species, antioxidant metabolism, crop protection, stress management strategies

## Abstract

Drought is one of the most important abiotic stresses responsible for reduced crop yields. Drought stress induces morphological and physiological changes in plants and severely impacts plant metabolism due to cellular oxidative stress, even in C4 crops, such as sugarcane. Seaweed extract-based biostimulants can mitigate negative plant responses caused by drought stress. However, the effects of foliar application of such biostimulants on sugarcane exposed to drought stress, particularly on plant metabolism, stalk and sugar yields, juice purity, and sugarcane technological quality, have received little attention. Accordingly, this study aimed to evaluate the effects of foliar application of a seaweed extract-based biostimulant on late-harvest sugarcane during the driest period of the year. Three experiments were implemented in commercial sugarcane fields in Brazil in the 2018 (site 1), 2019 (site 2), and 2020 (site 3) harvest seasons. The treatments consisted of the application and no application of seaweed extract (SWE) as a foliar biostimulant in June (sites 2 and 3) or July (site 1). The treatments were applied to the fourth ratoon of sugarcane variety RB855536 at site 1 and the fifth and third ratoons of sugarcane variety SP803290 at sites 2 and 3, respectively. SWE was applied at a dose of 500 ml a.i. ha^−1^ in a water volume of 100 L ha^−1^. SWE mitigated the negative effects of drought stress and increased stalk yield per hectare by up to 3.08 Mg ha^−1^. In addition, SWE increased stalk sucrose accumulation, resulting in an increase in sugar yield of 3.4 kg Mg^−1^ per hectare and higher industrial quality of the raw material. In SWE-treated plants, Trolox-equivalent antioxidant capacity and antioxidant enzyme activity increased, while malondialdehyde (MDA) levels decreased. Leaf analysis showed that SWE application efficiently improved metabolic activity, as evidenced by a decrease in carbohydrate reserve levels in leaves and an increase in total sugars. By positively stabilizing the plant’s cellular redox balance, SWE increased biomass production, resulting in an increase in energy generation. Thus, foliar SWE application can alleviate drought stress while enhancing sugarcane development, stalk yield, sugar production, and plant physiological and enzymatic processes.

## Introduction

Climate change and the global migration of agricultural production to non-traditional areas of cultivation are placing increasing pressure on modern agriculture. Abiotic stresses, such as drought or extremes of light or temperature, limit agricultural productivity (Ashraf and Foolad, 2007) and can reduce crop yields by as much as 50% (Vinocur and Altman, 2005; Qin et al., 2011; Vianna and Sentelhas, 2014; Voss-Fels and Snowdon, 2016). Sugarcane is an economically important crop that is cultivated in a wide range of edaphoclimatic environments, and foliar spray is commonly used as a management tool under conditions of abiotic stress. In the Brazilian production context, water availability in the soil is the abiotic factor with the greatest impact on yield and metabolic processes. Low water availability negatively affects the evapotranspiration rate, tillering, and leaf area of sugarcane, inducing senescence and reducing the stalk growth rate and crop development (Inman-Bamber, 2004; Inman-Bamber and Smith, 2005).

At the cellular level, plant responses to stress conditions include changes in the content of chlorophyll pigments (Cha-Um et al., 2012; Banerjee and Roychoudhury, 2019); cellular osmotic adjustment (Melo-Abreu and Ribeiro, 2010; Patade et al., 2011); early stomatal closure (Van Heerden et al., 2004; De Almeida Silva et al., 2013; Zhao et al., 2013); decreased quantum efficiency of photosystem II (Takahashi and Badger, 2011; Banerjee and Roychoudhury, 2019); and production of reactive oxygen species (ROS), which weakens cellular redox homeostasis in favor of oxidizing molecules and results in oxidative stress (Pinciroli et al., 2019). The main molecules responsible for oxidative stress are superoxide anion (O_2_^−^), singlet oxygen (^1^O_2_), hydrogen peroxide (H_2_O_2_), and hydroxyl radical (OH^−^), which are primarily produced in chloroplasts, mitochondria, and peroxisomes due to the dependence on O_2_ of the corresponding metabolic processes of aerobic respiration, photosynthesis, and photorespiration (Barbosa et al., 2014). These oxidants induce damage to proteins, nucleic acids, and photosynthetic pigments, in addition to activating programmed cell death and causing lipid peroxidation of membranes (Choudhury et al., 2017).

The European Biostimulant Industry Consortium (EBIC) defines “biostimulants” as substances that promote plant nutrition by improving the availability and absorption of nutrients from the soil and enabling greater tolerance of abiotic stresses (du Jardin, 2015). Biostimulant products can also be scientifically defined as “any substance or microorganism applied to plants with the aim to enhance nutrition efficiency, abiotic stress tolerance and/or crop quality traits, regardless of its nutrients content” (du Jardin, 2015) or “a formulated product of biological origin that improves plant productivity as a consequence of the novel, or emergent properties of the complex of constituents, and not as a sole consequence of the presence of known essential plant nutrients, plant growth regulators, or plant protective compounds” (Yakhin et al., 2017). Biostimulants are also referred to as biogenic stimulators, organic biostimulants, biostimulators, metabolic enhancers, and biostimulant plant growth promoters, among other terms (Yakhin et al., 2017). The use of the term biostimulants can be traced to 1951 (Yakhin et al., 2017); however, the potential of biostimulants to alleviate the negative effects of global climate change has been investigated in the last 25 years (Craigie, 2010; Sharma et al., 2014; Yakhin et al., 2017; Ricci et al., 2019; Jindo et al., 2020; Rai et al., 2021). Abiotic stresses, such as drought, are becoming increasingly important threats to food production due to recent shifts in temperatures (extreme temperatures) and weather patterns. Under these adverse environmental conditions, biostimulant application can alter plant metabolism and promote high plant performance (Huang et al., 2013; Feitosa De Vasconcelos et al., 2019) and may be an alternative management practice to ameliorate abiotic stress (Van Oosten et al., 2017; Yakhin et al., 2017; Shukla et al., 2019).

The term “biostimulant” is not found in Brazilian legislation, but Decree no. 4.954/2004 categorizes products that contain components with beneficial and stimulating effects on agricultural crops. These substances are classified as “biofertilizers defined as products that contain an active principle or organic agent, free from agrotoxic substances, that is capable of acting, directly or indirectly, on all or part of cultivated plants to increase their productivity, without taking into account its hormonal or stimulating value” (Art.2°. I; Lopes, 2021). Brazilian Normative Instruction No. 61, July 8th, 2020, establishes rules on definitions, specifications, guarantees, tolerances, registration, packaging, and labeling of organic fertilizers and biofertilizers intended for agriculture. The seaweed extract used in the present study is classified by the manufacturer as a Class “A” organomineral. The organomineral fertilizers in Art. 3° are categorized according to the raw materials used in their production. The category Class “A” includes products whose raw materials were generated in extractive, agricultural, industrial, agroindustrial, and commercial activities. These materials include mineral, vegetable, animal, industrial, and agro-industrial sludge from wastewater treatment systems whose responsible use is authorized by the Environmental Agency as well as pre- and post-consumption fruit, vegetable, and food waste segregated at the generating source and collected by differentiated collection. Such materials can be safely used in agriculture if they are free from waste or sanitary contaminants (Instrução normativa no 61, de 8 de julho de 2020—DOU—Imprensa Nacional, n.d.).

Foliar or soil application of biostimulants has been adopted for several crops to improve plant physiology and metabolism (Mariani and Ferrante, 2017), including in organic agriculture (Mógor et al., 2008). Seaweed extracts are the fastest growing category of biostimulants (Goñi et al., 2018) and have been shown to improve the drought tolerance of agricultural crops (Craigie, 2010; Sangha et al., 2014). Most seaweed species used to produce biostimulant extracts are classified as brown algae, most notably *Ascophyllum nodosum* (Craigie, 2010). However, green or red algae species can also be used as a source of raw material (Goñi et al., 2018). A biofertilizer comprising algae extracts or processed algae is a product obtained by extracting and processing algae (Instrução normativa no 61, de 8 de julho de 2020—DOU—Imprensa Nacional, n.d.). Seaweed extract may also be labeled as a biofertilizer (Zodape, 2001). Although Brazilian legislation does not apply the word biostimulant to seaweed extract, recent international scientific studies classify seaweed extracts, including extracts of *A. nodosum*, as biostimulant substances (Pohl et al., 2019; Taskos et al., 2019; González-González et al., 2020; Baltazar et al., 2021; do Rosário Rosa et al., 2021; Shukla and Prithiviraj, 2021). Seaweed extracts provide polysaccharides, polyunsaturated fatty acids, enzymes, bioactive peptides, LEA (late embryogenesis abundant) proteins, amino acids, plant hormones, and macro- and micronutrients (De Abreu et al., 2008; Shukla et al., 2016; Okolie et al., 2018). These extracts act on plant metabolism in a specific or non-specific manner (Carmody et al., 2020; Langowski et al., 2021). In general, seaweed extracts stimulate the synthesis of pigments, such as chlorophyll, to optimize photosynthesis, promote root growth and improve water and nutrient uptake, with direct effects on crop yields (Bulgari et al., 2015; Yakhin et al., 2017). Brown algae extract (*A. nodosum*) stimulates the activity of antioxidant enzymes and cellular accumulation of defense metabolites. Products with these characteristics typically protect and improve the response of the crop to water stress, thereby mitigating yield losses (Goñi et al., 2018; Jithesh et al., 2019; González-González et al., 2020).

In the present study, the effectiveness of a seaweed extract-based protective product on the physiology of sugarcane under drought stress and its implications for quality and stalk yields were evaluated. The hypothesis was that the application of a seaweed extract as a biostimulant *via* foliar application under drought stress would improve antioxidant activity in the sugarcane plant as well as raw material quality and biometric variables.

## Materials and Methods

### Experimental Area Description

Sugarcane (*Saccharum* spp. hybrids) field experiments were carried out under drought conditions in three different harvest seasons: site 1 (2018), site 2 (2019), and site 3 (2020) in fourth, fifth, and third sugarcane ratoons, respectively. The experiments were conducted during the drought season in the south-central region of Brazil from June to September. Site 1 is located in the area of the Bunge mill in Dourados, MS (22°0.13′0.18″S, 54°0.48′0.23″W). Site 2 is located in the area of the São Martinho mill in Pradópolis, SP (21°0.21′0.34″S, 48°0.03′0.56″W). Site 3 is located in the area of the São Martinho mill in Motuca, SP (21°0.30′0.30″S, 48°0.09′0.12″W). The average elevations are 448, 538, and 615 m asl at sites 1, 2, and 3, respectively. The soil was classified according to STAFF (2014), and soil chemical characteristics were determined before installation of the field experiments. At each site, 10 soil subsamples were collected from the experimental area between the ratoon rows and combined into one composite soil sample. The results of soil classification and chemical analysis for the experimental area are presented in Table 1.

**Table 1:** Soil classification and chemical characteristics of experiments at each site.

Site	Experiment	Soil classification	Depth	pH	M.O.	P_(resin)_	S	Al^+3^	H + Al^+3^	K	Ca	Mg	SB	CTC	V%
				CaCl_2_	g dm^−3^	mg dm^−3^	mmol_c_ dm^−3^								%
Site 1 Dourados (MS)	1	Rhodic Eutrudaf	0.00–0.20	5.2	29	32	37	0	35	2.2	66	18	86	121	71
			0.20–0.40	5.1	39	23	55	0	39	2.4	54	16	73	111	65
Site 2 Pradópolis (SP)	2	Rhodic Eutrudox	0.00–0.20	5.1	26	19	9	0	40	2.6	42	15	60	100	60
			0.20–0.40	5.1	23	16	29	0	42	1.7	33	15	49	91	53
Site 3 Motuca (SP)	3	Rhodic Hapludox	0.00–0.20	5.3	17	55	27	0	21	2.6	27	11	41	61	67
			0.20–0.40	5	14	34	33	1	25	0.8	17	6	24	49	49

**Figure 1:**
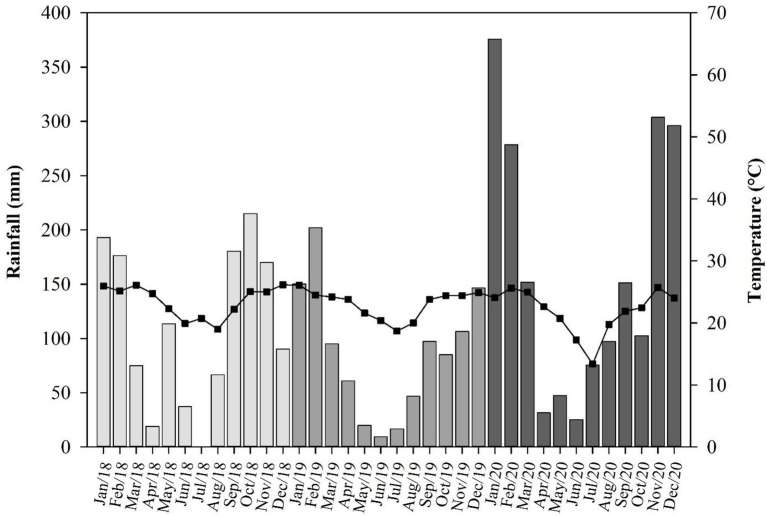
Monthly rainfall (bars) and mean temperatures (line/squares) during experimental period of 2018, 2019, and 2020 in sites 1, 2, and 3, respectively. Different colors of gray mean different years of experiment conduction.

According to the Köppen–Geiger climatic classification system, site 1 has a humid subtropical climate (Cfa), and sites 2 and 3 have a tropical savannah climate (Aw). At sites 1, 2, and 3, the annual average temperature is 21.3, 23.4, and 21.6°C, and the average precipitation is 1,700, 1,419, and 1,344 mm, respectively (Centro de Pesquisas Meteorológicas e Climáticas Aplicadas à Agricultura—CEPAGRI/UNICAMP, n.d.; Figure 1).

The sugarcane varieties were RB855536 (site 1) and SP803280 (sites 2 and 3). RB855536 has high agricultural and industrial productivity under favorable environmental conditions, with excellent ratoon regrowth, high tillering, high sucrose content, and medium to late ripening. SP803280 is characterized by high sucrose content and ratoon yield, moderate tillering, excellent ratoon regrowth, and medium to late ripening.

### Experimental Design and Treatments

**Figure 2:**
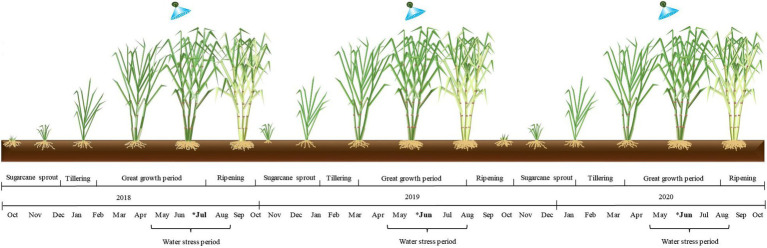
Schematic experimental timeline with descriptions of the stages of the growing seasons and the timing of seaweed extract biostimulant application in each growing season (*).

The experiment followed a randomized block coordinate (RBC) design comprising two treatments with 12 replications. The plots consisted of eight rows with a length of 10 m and an inter-row spacing of 1.5 m. The treatments were as follows: (1) control, no SWE application; and (2) SWE, application of a seaweed-based biostimulant extracted from *A. nodosum*. The SWE applications were performed in July at site 1 and in June at sites 2 and 3. All sites were harvested in October (Figure 2).

The SWE biostimulant was applied at a dose of 500 ml a.i. ha^−1^. The recommended dose per hectare is 0.5–1 L ha^−1^ for a water volume of 100 L ha^−1^. The product contained the following (in g/L): organic carbon = 78.0, N = 13.0, S = 40.3, B = 1.17, Co = 0.78, Fe = 16.9, Cu = 13.0, Mn = 14.3, Mo = 0.52, and Zn = 29.9. It is classified as a class A organomineral fertilizer and has a density of 1.30 g ml^−1^ at 20°C (FMC—Linha Fertís—Química do Brasil LTDA). The product classification may vary depending on country legislation, and thus, the SWE biostimulant may also be marketed as a biostimulant or organic foliar fertilizer.

In this work, based on European Union (EU) Regulation 2019/1009 of the European Parliament and of the Council of 5 June 2019, the SWE product was considered a biostimulant. In article 3, a “plant biostimulant” is defined as a product that stimulates plant nutrition processes independently of the product’s nutrient content with the sole aim of improving one or more of the following characteristics of the plant or plant rhizosphere: nutrient use efficiency; tolerance to abiotic stress; quality traits; and availability of confined nutrients in the soil or rhizosphere (Official Journal of the European Union, 2019).

All applications were performed under suitable environmental conditions using a pressurized sprayer (CO_2_) with a single 1/4KLC-9 brass fieldjet application tip coupled to a 2.6-m-long rod. This setup enabled simultaneous and homogeneous application in four plant rows, with an application range of 7.5 m. The working pressure was 344 kPa for a water volume of 100 L ha^−1^. The application and dosage of the products followed the manufacturer’s recommendations.

No problems with pests, weeds, or disease were observed in the experimental sites. Thus, crop management was carried out according to the recommendation for each site following the mill’s calendar for cultivation practices.

### Leaf Sampling for Metabolite and Enzyme Activity Analyses

The top visible dewlap (TVD) leaf or leaf +1 was collected according to Van Dlllewijn (1952). The tip and base of the leaf were discarded, and only the middle third of the leaf was used to evaluate hydrogen peroxide (H_2_O_2_) and malondialdehyde (MDA) content, Trolox-equivalent antioxidant capacity (TEAC), and superoxide dismutase (SOD; EC 1.15.1.1), catalase (CAT; EC 1.11.1.6), peroxidase (POD; EC 1.11.1.7), and polyphenol oxidase (PPO; EC 1.10.3.1) activity. Leaves were sampled between 9:00 and 10:00 am at 90 days after SWE application, prior to harvest. For enzymatic activity evaluations, the sampled tissue was quickly frozen in liquid nitrogen and stored at - 80°C in Falcon tubes.

#### Evaluation of Sugarcane Leaf Metabolites

Twenty leaves were collected and dried in an oven with forced-air circulation at 65°C for 72 h. The material was then ground in a Wiley mill using a sieve with a mesh size of 1 mm. These samples were used to measure total sugars, soluble sugars, starch, and sucrose contents, and the results were expressed as the % of each 100 g sample (Nelson, 1944; Somogyi, 1945).

#### Oxidative Stress and Antioxidant Enzymes

To calculate MDA content, lipid peroxidation was evaluated according to the method of Heath and Packer (1968). A molar extinction coefficient of 155 mM L^−1^ was used, and the results were expressed in nanomoles of MDA per gram of fresh weight. Before enzymatic analysis, the total protein content of the sample was determined according to the methodology described by Bradford (1976). H_2_O_2_ content was determined (Alexieva et al., 2001) by reference to a calibration curve and expressed in μmol g^−1^ of fresh weight (FW).

SOD activity was evaluated as described by Giannopolitis and Ries (1977) and expressed in units (U) of SOD per gram of protein. CAT activity was evaluated as described by Havir and McHale (1987) and expressed in nanomoles per minute per milligram of protein. POD activity was evaluated according to Allain et al. (1974) and expressed in micromoles of H_2_O_2_ per minute per gram of FW. PPO activity was evaluated according to Kar and Mishra (1976) and expressed in μmol of transformed catechol per minute per gram of FW.

Relative antioxidant capacity was measured as TEAC using 2,2-diphenyl-1-picrylhydrasil (DPPH) according to Ozgen et al. (2006) and expressed in milligrams of TE per gram of FW.

### Sugarcane Biometric Evaluations

To evaluate sugarcane development, the biometric parameters of 20 stalks randomly collected from each plot at the ripening phenological stage prior to harvest were determined. The following parameters were measured: (1) plant height (m), measured from the distance from the ground to the auricular region of the leaf +1, and (2) stalk diameter (using a digital caliper; Marafon, 2012).

### Sugarcane Technological Evaluations

At the same time as the biometric evaluations, 10 stalks were cleared at the height of the apical bud, defoliated, and sent to the mill’s PCTS laboratory to analyze the following characteristics according to the methodology defined in the Sucrose Content-Based Sugarcane Payment System and in accordance with Consecana’s semiannual updates of the technological evaluations described by (Fernandes, 2003): sucrose (%; sucrose concentration in the FW of stalk), fiber (%; dry water-insoluble matter in the sugarcane), purity (%; sucrose present in the total solids content in cane juice), total reducing sugar (TRS; all forms of sugars in sugarcane in the form of reducing or inverted sugars), reducing sugars (RS %; reducing substances in cane and sugar products calculated as invert sugar, predominantly hexoses).

### Stalk and Sugar Yields

A useful area in each plot was defined by two planting lines with a length of 2 m each. Stalk mass was evaluated at the ripening phenological stage prior to harvest. The mass of stalks present in the 4 m linear row was extrapolated to obtain the stalk yield in Mg ha^−1^. The sugar yield (Mg ha^−1^) was then calculated by multiplying the stalk yield (Mg ha^−1^) by TRS and dividing by 1,000.

### Biomass and Energy Production

Fiber and stalk yield were used to calculate bagasse at 50% moisture, and trash yield was calculated considering 140 kg of trash per Mg of stalks and 60% collection from the soil surface (Hassuani, 2005). Energy production was calculated considering that 1 Mg of trash has 4.96 MWh of primary energy and 1 Mg of bagasse has 4.94 MWh of primary energy (1 MWh = 3,600.00 MJ; Hassuani, 2005).

### Statistical Analysis

For all data, normality was first assessed using the Shapiro–Wilk test, and homoscedasticity was checked with Levene’s test. The data were then submitted to analysis of variance (ANOVA) by the *F*-test (*p* < 0.10) and analyzed using Fisher’s protected last significant difference (LSD) test; *p* < 0.10 was considered significant.

## Results

**Figure 3:**
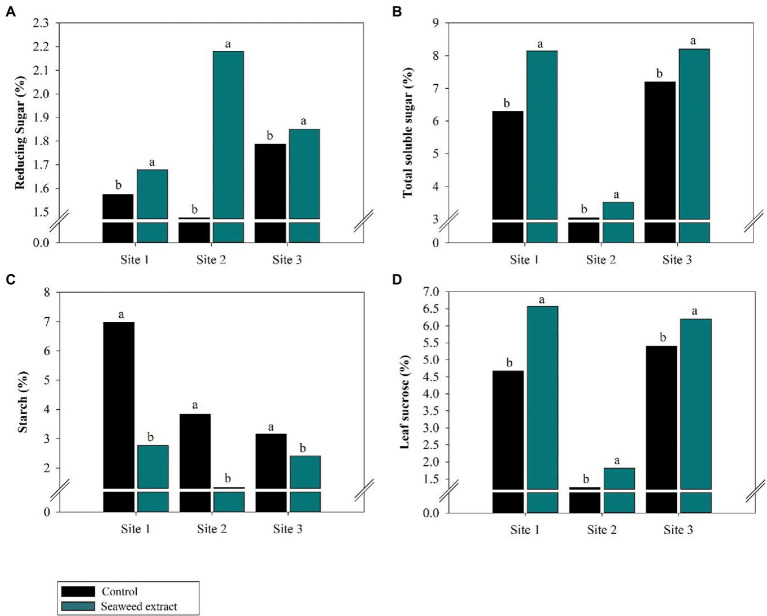
Effects of seaweed extract (SWE) application on sugarcane leaf metabolic parameters at harvest: **(A)** reducing sugars (%), **(B)** total soluble sugars (%), **(C)** starch (%), and **(D)** leaf sucrose (%). The treatments were as follows: Control, no SWE application; Seaweed extract: application of SWE biostimulant based on *Ascophyllum nodosum* in the beginning of the drought season (July at site 1 and June at sites 2 and 3). Averages followed by the same letters do not differ by the LSD test (*p* < 0.10).

At all sites, leaf metabolite levels were influenced by biostimulant application (Figure 3). Compared with the control, the SWE treatment significantly increased (*p* < 0.10) reducing sugars by an average of 35% and total soluble sugar by an average of 21% at all sites (Figures 3A,B). Leaf sucrose was also higher in the SWE treatment, with gains of 40.6, 45.6, and 13.1% at sites 1, 2, and 3, respectively, compared with the control (Figure 3D).

By contrast, starch levels were lower in the SWE treatment than in the control. At sites 1, 2, and 3, the starch content was 2.77, 1.50, and 2.50% in the SWE treatment and 6.98, 3.85, and 3.14% in the control, respectively (Figure 3C).

**Figure 4:**
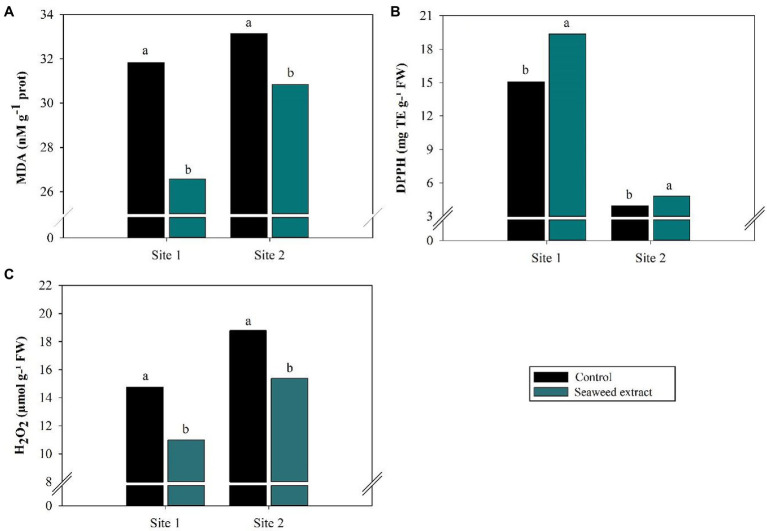
Effects of seaweed extract (SWE) application on sugarcane reactive oxygen species (ROS) scavenging parameters at harvest: **(A)** MDA (nM g^−1^ prot), **(B)** DPPH (mg trolox/100 g FW) and **(C)** H_2_O_2_ (μmol g^−1^ FW). The treatments were as follows: Control, no SWE application; Seaweed extract: application of SWE biostimulant based on *Ascophyllum nodosum* in the beginning of the drought season (July at site 1 and June at sites 2 and 3). Averages followed by the same letters do not differ by the LSD test (*p* < 0.10).

At the sites where it was evaluated (sites 1 and 2), the reactive potential of MDA, a marker of oxidative stress, was lower in the SWE treatment than in the control, with values of 31.82 nM g^−1^ prot and 33.15 nM g^−1^ prot in the control treatment but 26.57 nM g^−1^ prot and 30.83 nM g^−1^ prot in the SWE treatment (Figure 4A). Accordingly, TEAC (DPPH), which reflects antioxidant capacity, was 28.3 and 21.7% higher in the SWE treatment at sites 1 and 2, respectively, than in the control (15.1 mg TE g^−1^ FW and 3.95 mg TE g^−1^ FW, respectively; *p* < 0.10; Figure 4B). Leaf H_2_O_2_ levels were also significantly lower (*p* < 0.10) in the SWE treatment, with drops of 25.5 and 18.2% from the values of 14.8 μmol g^−1^ FW and 18.8 μmol g^−1^ FW in the control at sites 1 and 2, respectively (Figure 4C).

**Figure 5:**
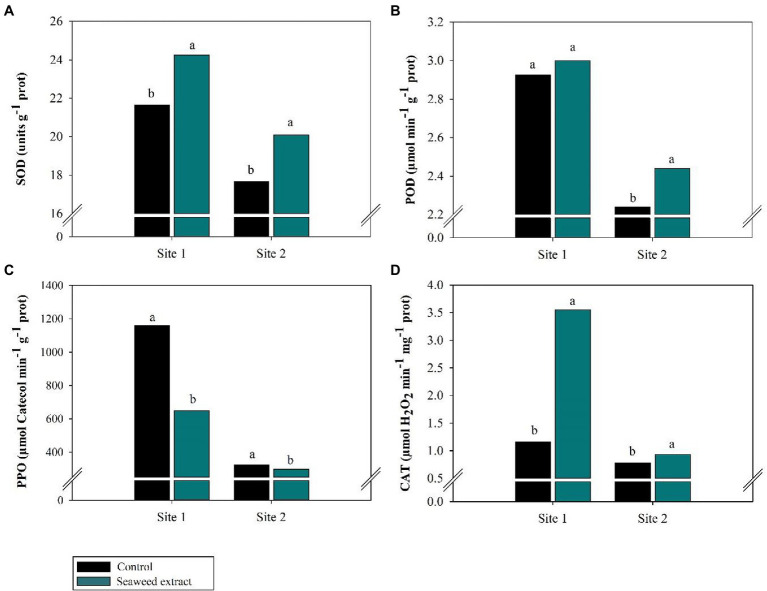
Effects of seaweed extract (SWE) application on sugarcane antioxidant enzymatic parameters at harvest: **(A)** SOD (units g^−1^ prot), **(B)** POD (μmol min^−1^ g^−1^ prot), **(C)** PPO (μmol Catechol min^−1^ g^−1^ prot), **(D)** CAT (μmol H_2_O_2_ min^−1^ mg^−1^ prot). The treatments were as follows: Control, no SWE application; Seaweed extract: application of SWE biostimulant based on *Ascophyllum nodosum* in the beginning of the drought season (July at site 1 and June at sites 2 and 3). Averages followed by the same letters do not differ by the LSD test (*p* < 0.10).

Increased (*p* < 0.10) activity of the antioxidant enzymes SOD, CAT, and POD was observed in the SWE treatment at both sites (Figure 5). At site 1, the application of SWE increased SOD and POD activity by 11.9 and 2.3%, respectively, compared with the control (21.7 units g^−1^ prot and 2.93 μmol min^−1^ g^−1^ prot, respectively). At site 2, SOD and POD activities were 13.8 and 8.9% higher, respectively, in the SWE treatment than in the control (17.66 units g^−1^ prot and 2.24 μmol min^−1^ g^−1^ prot, respectively; Figures 5A,B). CAT activity increased from 1.2 (site 1) and 0.78 μmol H_2_O_2_ min^−1^ mg^−1^ prot (site 2) in the control to 3.6 (site 1) and 0.93 μmol H_2_O_2_ min^−1^ mg^−1^ prot (site 2) in the SWE treatment, respectively (Figure 5D). On the contrary, the activity of PPO, an enzyme with oxidizing activity that causes the sample to darken in the presence of hydrogen peroxide (H_2_O_2_), was 43.9 and 8.2% lower in the SWE treatment at sites 1 and 2, respectively, compared with values of 1,158 and 323.16 μmol catechol min^−1^ g^−1^ prot in the control (Figure 5C).

**Figure 6:**
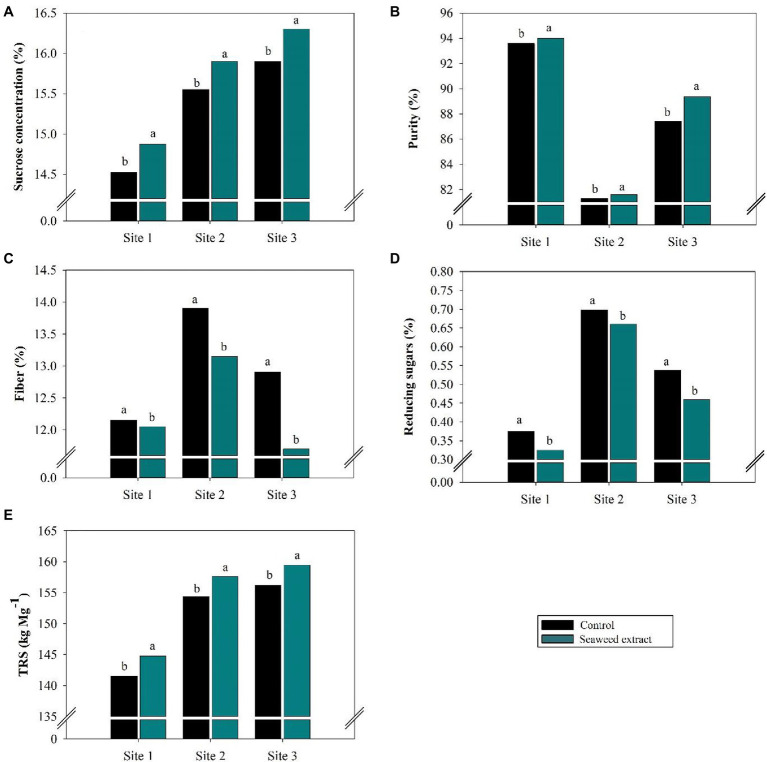
Effects of seaweed extract (SWE) application on sugarcane technological parameters at harvest: **(A)** sucrose concentration (%), **(B)** purity (%), **(C)** fiber (%), **(D)** reducing sugars (%), and **(E)** total reducing sugars (kg Mg^−1^). The treatments were as follows: Control, no SWE application; Seaweed extract: application of SWE biostimulant based on *Ascophyllum nodosum* in the beginning of the drought season (July at site 1 and June at sites 2 and 3). Averages followed by the same letters do not differ by the LSD test (*p* < 0.10).

The sucrose concentration (%) in the control was 14.52, 15.55, and 15.90% at sites 1, 2, and 3, respectively, and application of the SWE biostimulant significantly (*p* < 0.10) increased the sucrose concentration by 2.7, 2.6 and 2.6%, respectively (Figure 6A). As the sucrose concentration increased, the level of reducing sugars decreased at all sites (Figure 6D).

The juice purity (%) was greater in the SWE treatment than in the control (Figure 6B). Thus, SWE application improved the industrial quality of the raw material, with juice purity values of at least 80% at all sites. In contrast to the changes in sucrose concentration and juice purity, SWE application decreased fiber (%; Figure 6C). The fiber value was lowest when SWE was applied at site 3, with a decrease of 9.3% relative to the control (12.69%).

The sucrose concentration and level of reducing sugars were used to calculate TRS (Figure 6E). Consistent with the significant influence (*p <* 0.10) of the SWE treatment on the sucrose concentration, SWE application increased TRS by an average of 3.2 kg Mg^−1^ at sites 1 and 2 and 3.3 kg Mg^−1^ at site 3. The TRS values in the control were 141.6, 154.4, and 156.2 kg Mg^−1^ at sites 1, 2, and 3, respectively.

**Figure 7:**
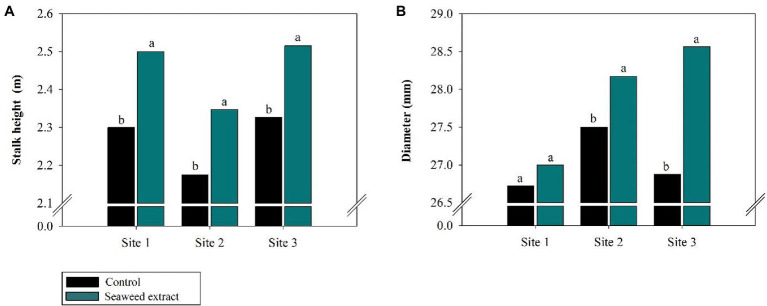
Effects of seaweed extract (SWE) application on sugarcane biometric parameters at harvest: **(A)** stalk height (m), **(B)** diameter (mm). The treatments were as follows: Control, no SWE application; Seaweed extract: application of SWE biostimulant based on *Ascophyllum nodosum* in the beginning of the drought season (July at site 1 and June at sites 2 and 3). Averages followed by the same letters do not differ by the LSD test (*p* < 0.10).

**Figure 8:**
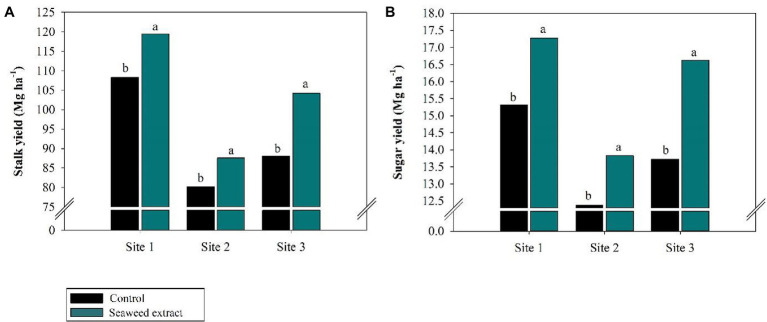
Effects of seaweed extract (SWE) application on sugarcane yield parameters at harvest: **(A)** stalk yield (Mg ha^−1^), **(B)** sugar Yield (Mg ha^−1^). The treatments were as follows: Control, no SWE application; Seaweed extract: application of SWE biostimulant based on *Ascophyllum nodosum* in the beginning of the drought season (July at site 1 and June at sites 2 and 3). Averages followed by the same letters do not differ by the LSD test (*p* < 0.10).

Among the biometric parameters, stalk height was positively affected (*p* < 0.10) by SWE application at all sites (Figure 7A), with an average gain of 0.2 m at sites 1 and 3 and 0.18 m at site 2 compared with the control (2.30, 2.33, and 2.18 m at sites 1, 2, and 3, respectively). Furthermore, stalk diameter was larger in the SWE treatment at all sites except at site 1, where no significant difference was observed between the treatments (Figure 7B). SWE application significantly increased stalk yield, with average gains of 10.2, 9.2, and 18.3% at sites 1, 2, and 3, respectively, relative to the control (108.4, 80.2, and 88.07 Mg ha^−1^, respectively; Figure 8A). As sugar yield is the product of TRS and stalk yield, increases in the latter parameters will directly increase sugar yield. At sites 1, 2, and 3, sugar yield was 13.1, 11.3, and 20.9% higher in the SWE treatment than in the control (15.3, 11.3, and 20.9 Mg ha^−1^, respectively; Figure 8B).

**Figure 9:**
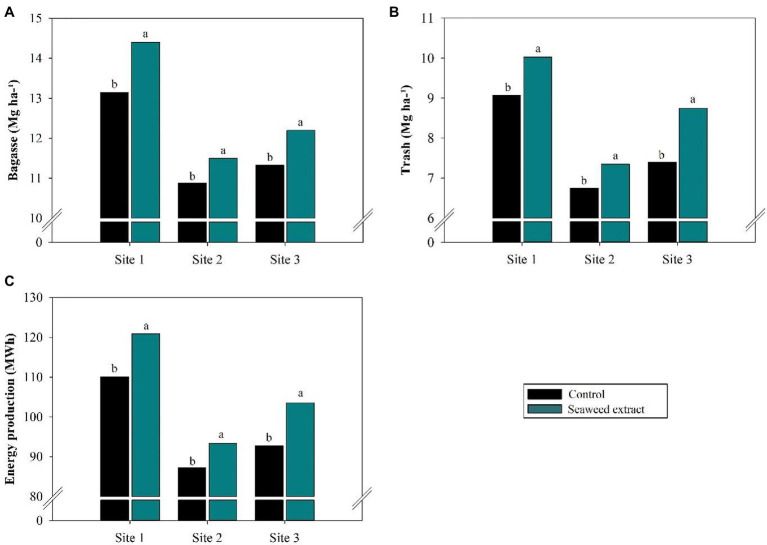
Effects of seaweed extract (SWE) application on sugarcane biomass and energy parameters at harvest: **(A)** bagasse (Mg ha^−1^); **(B)** trash (Mg ha^−1^) and **(C)** energy production (MWh). The treatments were as follows: Control, no SWE application; Seaweed extract: application of SWE biostimulant based on *Ascophyllum nodosum* in the beginning of the drought season (July at site 1 and June at sites 2 and 3). Averages followed by the same letters do not differ by the LSD test (*p* < 0.10).

In general, the application of SWE increased biomass production (bagasse and trash) at all sites (Figure 9). Bagasse production was highest in the SWE treatment at site 1, with an increase of 1.2 Mg ha^−1^ compared with the control (13.2 Mg ha^−1^; Figure 9A). Trash production was highest at site 3, with a gain of 1.35 Mg ha^−1^ in the SWE treatment compared with the control (7.4 Mg ha^−1^; Figure 9B). Finally, energy production at sites 1, 2, and 3 was 9.8, 7.1, and 11.6% higher in the SWE treatment than in the control (110.1, 87.2, and 92.7 MWh, respectively; Figure 9C).

## Discussion

Plants adopt a variety of management strategies to maintain physiological water balance, including increased water uptake from the soil, stomatal closure, osmotic adjustment in plant tissues, hormonal signaling of abiotic stress, and production of antioxidant species and defense metabolites (Gupta et al., 2020). The efficiency of the plant response to stress factors is inextricably linked to stress severity, stress duration, number of exposures, combinations of stresses, and even the plant’s genetic composition. Extreme changes in environmental patterns may induce irreversible stress conditions in plants, reducing their phenological plasticity and yield (Hauvermale and Sanad, 2019). Here we evaluated the ability of a seaweed extract (SWE)-based biostimulant to mitigate drought stress in sugarcane plants. The use of SWE has been associated with significant increases in crop growth, yields, and resistance to abiotic stresses (Khan et al., 2009).

Under drought stress conditions, the initial conversion of sugars into starch is desirable to avoid a high sugar concentration, which will inhibit photosynthesis and generate a senescence response (Paul and Foyer, 2001; Rosa et al., 2009) due to deficient solute transport in the phloem, enabling loading from source (leaves) to sink (stalks; Yamada et al., 2010; Lemoine et al., 2013; Gong et al., 2015). The greater accumulation of starch in leaves in the control (Figure 3C) resulted in a lower rate of sugar synthesis in the cell medium. When the solute concentration in the medium decreases, the guard cells in the stomata increase their water potential due to the greater free energy of water, which in turn reduces cell turgor and closes the stomata. Stomatal closure reduces excessive water loss to the atmosphere and improves the drought stress response (Valerio et al., 2011; Prasch et al., 2015) but directly reduces plant yield. However, the role of starch in plant metabolism is plastic, and the response of starch to stress factors varies. Thus, starch cannot be considered only a reserve carbohydrate. The starch response depends on the type of plant tissue and its function in the plant, in addition to the source–sink relation (Thalmann and Santelia, 2017; Dong and Beckles, 2019).

Our findings revealed a rapid response of the plant to drought that involved starch metabolism, as evidenced by the effects of the application of the SWE biostimulant. SWE application can improve water use efficiency and optimize energy and carbon use, even under limiting conditions for photosynthesis. Mobilizing this carbohydrate reserve into total derived sugars increases the drought tolerance of the plant and promotes conditions favorable for growth (Thalmann and Santelia, 2017), as sugars are substrates for protective proteins and the synthesis of other compounds that are essential for cell membrane protection (Taiz et al., 2017). The attenuation of abiotic stress mediated by the application of SWE likely increased plant consumption of starch reserves in leaves, resulting in the release of glucose, among other components, and an increase in the reducing sugar index in source tissues (Zeeman et al., 2010). Monosaccharides released in the leaf cytosol are used to synthesize sucrose *via* the activity of the enzyme SPS (Dong and Beckles, 2019), thereby enhancing total soluble sugar. In addition, the metabolites generated by starch degradation (Figures 3A,D) are essential for maintaining the osmoprotective balance of plant cells and cell volume, which prevents plants from losing water to the extracellular medium (Thalmann and Santelia, 2017).

The application of SWE also affected enzyme activity (Figure 5B). In plants treated with SWE, the activity of the enzyme PPO decreased. This enzyme is concentrated in plastids, where it participates in the control of oxygen levels in the region of photosystems I and II to promote ROS balance for healthy cell function, that is, photosynthesis, under stress conditions (Thipyapong et al., 2004; Boeckx et al., 2015). PPO is involved in the browning of sugarcane juice, which is undesirable in the sugar industry (Vaughn and Duke, 1984; Vickers et al., 2005; Naikoo et al., 2019). Browning is caused by the reaction between the enzyme and phenolic compounds (flavonoids, tannins, hydroxycinnamate esters, and lignin). These phenolic compounds are secondary metabolites that are synthesized in the cytosol and are involved in plant defense mechanisms against stress (Vaughn and Duke, 1984; Vickers et al., 2005; Naikoo et al., 2019). By relieving drought stress, SWE application likely contributed to the maintenance of enzyme levels, thereby decreasing the oxidation of phenolic compounds.

Positive effects of SWE treatment on cellular redox balance, ROS, and drought stress have previously been reported for bean (*Phaseolus vulgaris*), tomato (*Lycopersicon esculentum*), soybean (*Glycine max*), sweet orange (*Citrus sinensis*), and spinach (*Spinacea oleracea*; Spann and Little, 2011; Xu and Leskovar, 2015; Carvalho et al., 2018; Shukla et al., 2018). In a study of non-stressed plants, Goñi et al. (2016) observed increased plant expression of antioxidant system-related genes in the presence of *A. nodosum* extract. In the present study, the application of SWE increased antioxidant capacity as measured by TEAC-DPPH, enhanced radical scavenging activity in leaves, and contributed to increased cellular phenolic content levels, consistent with previous work (Fan et al., 2011; Spann and Little, 2011; Lola-Luz et al., 2014; Elansary et al., 2016). Therefore, exogenous application of SWE can enhance the activity of antioxidant enzymes (SOD, CAT, and POD) and molecules (α-tocopherol, ascorbate, and β-carotene; Allen et al., 2001; Shi et al., 2018; Yildiztekin et al., 2018; Shukla et al., 2019).

The decrease in ROS levels in the SWE treatments was associated with an increase in SOD activity (Figure 5A). SOD catalyzes the dismutation of two molecules of O_2_^−^ to form one molecule of H_2_O_2_ and one molecule of O_2_. By removing O_2_^−^, SOD reduces the risk of cellular OH^−^ formation (Bhattacharjee, 2010; Dubey and Pandey, 2011; Dinakar et al., 2012). SWE application also enhanced the activity of the enzymes POD and CAT (Figures 5B,D). These enzymes are crucial for defense against oxidative stress, as they convert the excess H_2_O_2_ generated by the enzymatic action of SOD into H_2_O (Ken et al., 2005; Ballesteros et al., 2009; Nascimento and Barrigossi, 2014). SWE has previously been reported to eliminate cellular H_2_O_2_ in plants and consequently contribute to stable redox state maintenance, alleviating drought stress (Caverzan et al., 2016; do Rosário Rosa et al., 2021).

Plants treated with SWE also exhibited reduced levels of the 3-carbon dialdehyde MDA (Figure 4A). MDA is produced when ROS oxidize polyunsaturated fatty acids in cell membranes, causing irreversible cell damage (Goñi et al., 2018; Shukla et al., 2019). The impact of MDA is greatest in organelles, such as chloroplasts and mitochondria, which have intense oxidative metabolisms and high concentrations of polyunsaturated fatty acids (Mano, 2012). SWE treatment of sugarcane under drought stress reduced MDA levels in plant cells. SWE likely neutralizes the oxidizing action of ROS in chloroplasts and mitochondria, which together are responsible for converting atmospheric carbon into substrates and metabolic energy through photosynthesis and cell respiration.

Because SWE contains plant hormones, it can act directly or indirectly on carbohydrate metabolism (Craigie, 2010; Ali et al., 2016) to affect raw material quality. Raw material quality is directly related to stalk accumulation of sucrose. Therefore, in addition to acting as a biostimulant, SWE may help sugarcane reach sucrose levels favorable for industrialization (Figure 6A), that is, equal to or greater than 13% (Deuber, 1988), under drought stress. As shown in Figure 6, the increase in the sucrose concentration in the SWE treatment led to improved technological parameters, including higher sugarcane juice purity. The decreases in glucose, fructose, and fiber levels resulted in higher total recoverable sugar (TRS). Other studies have also reported a strong influence of polysaccharides that are abundant in various seaweed extracts, including those from *A. nodosum*, on vegetative parameters (Craigie, 2010; Omidbakhshfard et al., 2020; Baltazar et al., 2021; Rasul et al., 2021). These non-growth hormone compounds modulate metabolic, lipid, and transcription pathways to promote phenotypic changes in plants that facilitate growth, such as improved resistance to stress, reduced ROS levels, and decreased electrolyte loss due to cell damage (Craigie, 2010; Omidbakhshfard et al., 2020; Baltazar et al., 2021; Rasul et al., 2021). These phytotonic effects of SWE arise from the complex composition of metabolites, which are rich in macro- and micronutrients, carbohydrates, amino acids, and plant hormones, such as auxins and cytokinin (De Abreu et al., 2008; Tan et al., 2021).

The plant growth-promoting complex found in SWE can induce cell division and expansion, enhance photosynthetic activity, contribute to the partitioning of metabolic energy and synthesis of biochemical components, and directly impact plant height, diameter, and stalk yields (Deshmukh and Phonde, 2013; Singh et al., 2018; Shukla et al., 2019; Diwen et al., 2021; Tan et al., 2021). Hormones found in SWE influence root growth (Rathore et al., 2009; Wozniak et al., 2020; Diwen et al., 2021) and induce the expression of genes linked to nutrient transport (Rathore et al., 2009; Krouk et al., 2010), thereby enhancing soil exploration and nutrient uptake by plants (Omidbakhshfard et al., 2020; Rasul et al., 2021). Our results suggest that the biostimulant effects of SWE significantly increased the vegetative parameters of sugarcane, particularly stalk height and diameter (Figures 7A,B). The increase in stalk yields was likely due to the improved efficiency of primary metabolism under drought stress conditions (Figure 8A). As measures of productivity, the increases in TRS and stalk yield observed in this study confirmed that SWE satisfactorily improved the plant response under drought stress conditions. In turn, the increase in sugarcane productivity increased sugar yield (Figure 8B).

Bagasse and straw production are directly affected by vegetative variations in the plant induced by the phytotonic effects of SWE, as bagasse and straw are directly linked to stalk and fiber production. According to Hassuani et al. (2005), stalk yield and fiber at 50% humidity are used to calculate bagasse, while trash is calculated considering 140 kg of trash per Mg of stalk. The biostimulant activity of SWE in plants clearly promoted bagasse and straw production. In addition, increased biomass production directly improved the energy potential of plants treated with the SWE biostimulant, even under drought conditions.

In summary, this evaluation of SWE as a tool for mitigating drought stress in sugarcane demonstrated that SWE application can improve yields under unfavorable environmental conditions. The effects of SWE on plant physiology and enzymatic activity protect metabolic processes and promote the metabolism of carbohydrates essential for plant growth and increased biomass production per area.

## Conclusion

Seaweed extract, a protective product based on algae extract, effectively improved sugarcane stalk yield and quality under drought stress in this study. SWE enhanced the physiological, metabolic, and enzymatic responses of sugarcane and stimulated carbon assimilation and carbohydrate metabolism. SWE improved antioxidant activity and carbohydrate synthesis in the plant, leading to lower reducing sugar and fiber content and greater juice purity. SWE also enhanced the biometric parameters of sugarcane under drought stress, with taller and thicker stalks and, consequently, higher stalk and sugar yields and a metabolically stronger plant. Further investigation is required to establish the effectiveness of SWE across a complete sugarcane cycle and in the long term.

## Data Availability Statement

The original contributions presented in the study are included in the article/supplementary material, further inquiries can be directed to the corresponding author.

## Author Contributions

LJ and GS had contributed to conceptualization, data acquisition, data analysis, and design of methodology. LJ, JV, MO, LM, and CC wrote and edited the manuscript. All authors contributed to the article and approved the submitted version.

## Funding

This study was funded by CAPES, Coordination of higher level personal improvement.

## Conflict of Interest

The authors declare that the research was conducted in the absence of any commercial or financial relationships that could be construed as a potential conflict of interest.

## Publisher’s Note

All claims expressed in this article are solely those of the authors and do not necessarily represent those of their affiliated organizations, or those of the publisher, the editors and the reviewers. Any product that may be evaluated in this article, or claim that may be made by its manufacturer, is not guaranteed or endorsed by the publisher.
